# Specialized short term crisis intervention for patients with personality disorder: Effects on coercion and length of stay

**DOI:** 10.1177/00207640241277161

**Published:** 2024-09-04

**Authors:** Lisa L. Dinsenbacher, Lukas Imfeld, Fabrice Helfenstein, Julian Moeller, Undine E. Lang, Christian G. Huber

**Affiliations:** 1 University Psychiatric Clinics (UPK), University of Basel, Switzerland; 2 Faculty of Medicine, University of Basel, Switzerland; 3 Clinical Trial Unit, Department of Clinical Research, Faculty of Medicine, University of Basel, Switzerland; 4 Division of Clinical Psychology and Epidemiology, Department of Psychology, University of Basel, Switzerland

**Keywords:** Personality disorders, crisis intervention, coercion, length of stay

## Abstract

**Background:**

Acute crises in patients with personality disorders (PD) are often accompanied by suicidal and self-harming behavior. Their management is challenging, as both coercive measures and prolonged inpatient-treatment are known to be counterproductive. Only in crises that cannot be controlled by outpatient means, inpatient treatment is to be taken into account. This treatment should be time-limited and not involve coercion.

**Aims:**

The aim of this study was to assess if the introduction of a specialized crisis intervention track is associated with a reduction of coercive measures as well as a shorter in-hospital stay in PD patients.

**Methods:**

In this 8-year, hospital-wide, longitudinal, observational study, we investigated the frequency of coercive measures and the median length of in-hospital stay in 1,752 inpatient-cases with PD admitted to the Adult Psychiatry, UPK, Basel, Switzerland, between 01.01.2012 and 31.12.2019. By means of an interrupted-time-series analysis, we compared the period before and after the implementation of a specialized crisis intervention track for PD patients.

**Results:**

Our data show a significant decrease in the median length of in-hospital stay and no significant reduction in the incidence rate of coercion among PD patients after the intervention. The latter is likely due to a floor effect, since there was a significant decrease in coercive measures over the entire observation period, already reaching very low rates before the intervention.

**Conclusions:**

Our study underlines the clinical importance of specialized short-term crisis management in PD, which comes along with shorter lengths of in-hospital stays and a stable low rate of coercive measure.

## Introduction

Personality disorders (PD) pose special challenges to the therapist-patient relationship and to the therapeutic setting. This is, among other things, due to the fact that PD patients often have a reduced ability to form alliances and at the same time are at high risk for self-injurious behavior and suicidality. Both factors tend to cause emergency inpatient treatment—not infrequently against the patient’s will and with the help of coercive measures (Steinert et al., 2007; Steinert et al., 2008). Among all PD, Borderline personality disorder (BPD) is considered the most relevant for acute psychiatry: firstly, because it is disproportionately common within emergency psychiatry and inpatient clinical settings—both compared to other PD and to the BPD prevalence in the general population (Gunderson et al., 2013). Secondly, because the therapeutic approach can be particularly demanding. For PD patients it is well known that both long-term inpatient crisis intervention and coercive measures are not effective and even bear considerable risks: in the short term, they can have an escalating rather than deescalating impact. In the long term, they lead to chronic conditions that are difficult to treat, for example, chronic suicidality (Gunderson, 2009; Paris, 2004). This is why outpatient treatment settings for PD patients are state of the art and inpatient crisis interventions should only be considered in acute crises that overburden the outpatient treatment system. These interventions should—in line with the recommendations of the Swiss Society for Psychiatry and Psychotherapy—be limited to a maximum duration of 2 weeks, refrain from coercive measures even in cases of suicidality or self-injurious behavior and aim at a rapid reintegration into the outpatient treatment setting (Euler et al., 2018). In order to meet the diagnosis-related needs of its patients, the Department of Adult Psychiatry, Universitäre Psychiatrische Kliniken (UPK) Basel, University of Basel, Switzerland, has been pursuing a track concept with disorder-specific wards and treatment paths since 2012 (Lang et al., 2022). In this context, and specifically, although not exclusively, for PD patients, the concept of crisis intervention wards has gradually been gaining importance. Crisis intervention wards follow an open-door policy and allow overnight stay during a limited time period. They aim at stabilizing a person in psychiatric crisis, at shortening the length of stay as compared to a standard acute psychiatric admission and, finally, at reducing the need for further mental health care presentation (Anderson et al., 2022). To the authors’ knowledge, there is little literature and no substantial evidence to date regarding the influence of crisis intervention wards on the frequency of coercive measures and on the average length of in-hospital stay in PD patients.

The aim of the present study was to examine the influence of the implementation of a diagnosis-related crisis track concept for patients with PD on the frequency of coercive measures (primary objective) and on the length of in-hospital stay (secondary objective) for these patients. In this context, our study represents a program evaluation. We hypothesize that the incidence rate of seclusion and/or forced medication as well as the median length of in-hospital stay decrease with the implementation of a specialized crisis intervention track for patients with PD.

## Methods

### General framework and study design

The Department of Adult Psychiatry, Universitäre Psychiatrische Kliniken (UPK) Basel, University of Basel, Switzerland, provides inpatient and outpatient treatment for all psychiatric diagnoses. It has a healthcare mandate for all persons in Basel-City and the surrounding area, which covers a population of approximately 200,000 people, and basic healthcare insurance does not include inpatient treatment in other cantons. The UPK Basel has implemented a clinic-wide change to an open-door psychiatry with a minimum of coercive measures starting in 2010 (Hochstrasser et al., 2018; Huber et al., 2016; Lang et al., 2016; Lang et al., 2017) and has introduced a track concept that helps to directly admit persons to a ward specialized for their psychiatric disorder, providing integrated psychiatric and psychotherapeutic treatment from day one, even in acute psychiatry settings (Deuschle et al., 2020; Hirjak et al., 2020; Lang et al., 2022; Sollberger & Lang, 2013; Sollberger & Lang, 2014).

Data was documented during routine treatment and anonymized during data extraction. Our study was performed in accordance with all national and international legal regulations and with the Declaration of Helsinki in its current version. The presented analyses are part of a larger project on coercion and service use in psychiatry that was approved by the local ethics committee (Ethikkommission Nordwest- und Zentralschweiz, EKNZ 287-13 / PB_2020_ 00029).

### Short term crisis intervention ward

In 1991, a psychiatric crisis intervention ward (KIS) was established at the University Hospital Basel (USB), where most medical disciplines and the emergency room for the area are located. The KIS was designed for short (lasting a maximum of 5 days) and intensive psychiatric inpatient treatment in an open setting. Back then, it was composed of eight beds in four double rooms and intended for patients with a wide range of psychiatric disorders, such as adjustment disorders, psychosis, or depression. The KIS played a key role within the area’s suicide prevention strategy. As such, it was—at first—not part of the track system for diagnosis specific treatment as detailed above.

From January 2012 to September 2016, persons with personality disorders (PD) received planned mid- to long-term inpatient treatment on a specialized psychotherapeutic ward (PTA). Acute psychiatric inpatient treatment for PD patients was distributed over the other wards, for example, a long-term crisis intervention ward in the affective disorder track (Supplemental Table S2) and an acute intensive care ward (Supplemental Table S4) in the psychotic disorders track.

This system was perceived as suboptimal and not in-line with the established track system. Starting from October 2016, the KIS was therefore repurposed as a specialized short term crisis intervention ward for patients with PD. Due to a change of location at the USB, it was possible to increase the number of inpatient beds from eight beds in four double rooms to 12 beds in eight single rooms and two rooms that could be either used for as one-or two-bed rooms, and to increase the maximum treatment duration from 5 to 7 days. Patient-centered assessments have been integrated in the clinical routine and the interprofessional team consisting of psychiatrists, nurses, social workers, and further therapists, was specifically trained for PD crisis intervention and treatment, notably with advanced training in dialectical behavioural therapy, skills training, and regular case-based team supervision within the framework of an intensified, moderated team development process. A general conceptual change of the overall crisis intervention track for PD patients was established and since then PD patients have been preferably admitted to the new short-stay ward. It is one of a total of five specialized treatment tracks that are available (besides PD) also for psychotic disorders, substance use disorders, personality disorders, affective disorders, and geriatric psychiatry.

The present study compares the period before the new crisis intervention track for patients with PD was established (January 2012 to September 2016) with the period after the change (October 2016 to December 2019) and thus constitutes a program evaluation. Staff members were aware of clinical monitoring; however, they were not informed that a scientific evaluation of the data would be performed.

### Study population

Inclusion criteria for the present study were inpatient treatment for at least 24 hr at the Department of Adult Psychiatry, UPK Basel, during the observation period from 2012 to 2019, and a main diagnosis of a PD as defined by WHO ICD-10 (Organization, 1992). Patients under 18 years of age at admission as well as cases admitted before 2012 or dismissed after 2019 were excluded. No further inclusion or exclusion criteria were defined to ensure a naturalistic sample. The resulting dataset contained a total of 1,752 cases (see also Supplemental material S6).

### Statistical analysis

A statistical analysis plan was written and archived under version control prior to analyzing the data according to the standard operating procedures at the Department of Clinical Research, DKF Basel, Switzerland. The primary objective was to investigate whether and to what extent in a population of patients with PD admitted to the Department of Adult Psychiatry, UPK Basel, the incidence rate of seclusion and/or forced medication changed with the introduction of a new crisis intervention track for patients with PD on 01.10.2016. Our secondary objective was to examine whether the median length of in-hospital stay changed with the introduction of the new crisis intervention track. Accordingly, we defined the incidence rate of seclusion and/or forced medication per time period as the primary endpoint and the median length of in-hospital stay in hours per time period as the secondary endpoint.

For this study, we used an interrupted time series analysis (McDowall et al., 2019), which is a valuable design to assess the effectiveness of a population-level health intervention implemented at a clearly defined time point. We used the framework of segmented regression (also called piecewise regression or broken-stick regression) to investigate whether and how the intervention *introduction of a new crisis intervention track for patients with PD* induced an abrupt change, around a breakpoint, in the relationship first between time and the incidence rate of seclusion and/or forced medication and second between time and the median length of in-hospital stay. All analyses were performed in R version 4.2.0 (2022-04-22) (R Development Core Team, 2022; see also Supplemental material S7).

## Results

A total of 1,752 cases of patients with PD as their main diagnosis were admitted to and completed treatment at the Department of Adult Psychiatry, UPK Basel, during the observation period between 2012 and 2019.

Table 1 shows, on an annual basis, the overall bed occupancy, the total number of patients with PD as their main diagnosis, their clinical and sociodemographic characteristics (age at admission, gender), the type of admission (voluntary, involuntary), the average score for HoNOS item 1 (aggression/overactivity) and HoNOS item 2 (self-harm/suicidality), as well as the number of cases with coercive events (seclusion and/or forced medication) and the median length of in-hospital stay.

**Table 1.** Overall bed occupancy at admission and number of cases with PD main diagnosis at the department of adult psychiatry, UPK Basel, as well as clinical and sociodemographic characteristics of patients with PD main diagnosis (*n* = 1,752) per year.

Variables	2012	2013	2014	2015	2016	2017	2018	2019
Bed occupancy (%)	95.3%	97.4%	96.7%	96.5%	96.9%	95.1%	91.7%	94.1%
Number of cases with PD main diagnosis (*n*, %)	222 (7.7%)	218 (7.3%)	222 (7.6%)	190 (6.3%)	197 (6.4%)	195 (6.4%)	251 (8.2%)	257 (8.2%)
Age in years (mean ± *SD*)	34.0 ± 11.7	35.5 ± 12.0	33.1 ± 10.7	35.4 ± 14.0	34.2 ± 12.4	35.0 ± 12.8	34.6 ± 12.8	35.8 ± 14.1
Female gender (*n*, %)	154 (69.4%)	156 (71.6%)	149 (67.1%)	134 (70.5%)	128 (65.3%)	118 (60.5%)	161 (64.1%)	153 (59.5%)
Type of assignment								
Voluntary (*n*, %)	204 (91.9%)	202 (92.7%)	211 (95.0%)	179 (94.2%)	190 (96.4%)	190 (97.4%)	240 (95.6%)	248 (96.5%)
Involuntary (*n*, %)	18 (8.1%)	16 (7.3%)	11 (5.0%)	11 (5.8%)	7 (3.6%)	5 (2.6%)	11 (4.4%)	9 (3.5%)
HoNOS (mean ± SD)								
Item 1 score (aggression/overactivity)	1.6 ± 1.4	1.3 ± 1.4	1.2 ± 1.4	0.9 ± 1.3	0.9 ± 1.1	0.9 ± 1.2	0.8 ± 1.0	0.6 ± 0.8
Item 2 score (self-harm/suicidality)	1.1 ± 1.4	1.2 ± 1.4	1.1 ± 1.4	0.9 ± 1.4	0.8 ± 1.2	0.7 ± 1.2	1.3 ± 1.3	1.2 ± 1.2
Number of cases with seclusion and/or forced medication (*n*, %)	11 (5.0%)	6 (2.8%)	6 (2.7%)	4 (2.1%)	3 (1.5%)	3 (1.5%)	3 (1.2%)	3 (1.2%)
Length of stay in hours (median)	281.7	275.2	421.9	372.9	410.4	417.3	240.4	170.0

*Note.* Values are given as number (percentage) for nominal variables and in mean ± standard deviation for continuous variables.

From 2012 to 2019, the bed occupancy ranged from 91.7% to 97.4% and the number of cases with PD as main diagnosis from 190 to 257, both with no consistent trend over time. The mean age of our sample ranged from 33.1 to 35.8 years and there was a higher percentage of female than male subjects (ranging from 59.5% to 71.6% over the years). The percentage of involuntary admission decreased over the observation period. The average score for HoNOS item 1 (aggression/overactivity) declined between 2012 and 2019, while HoNOS item 2 (self-harm/suicidality) remained stable apart from some fluctuation. The number and percentage of seclusion and/or forced medication considerably decreased during the observation period. The median length of in-hospital stay ranged from 170.0 to 421.9 hr.

Table 2 shows the odds ratios associating the incidence of seclusion and/or forced medication with the intervention (factor: before/after introduction of the new crisis intervention track), the time period and their interaction, as well as seasonal variations (Fourier terms), the mean patient age and the sex ratio. There was no change in the incidence rate of seclusion and/or forced medication after the new crisis intervention track was introduced (Introduction of the new Crisis intervention track × Time; odds-ratio [95% CI] = 1.037 [0.776, 1.369], *p* = .798). Neither mean patient age at admission nor sex ratio contributed to explaining variation in the incidence rate of seclusion and/or forced medication.

**Table 2.** Generalized linear model investigating the variation in the incidence rate of seclusion and/or forced medication from 2012 to 2019. Effects of the explanatory variables are assessed as odds-ratios, that is, coefficients applying to the rate (probability) of the occurrence of seclusion and/or forced medication.

Variable	Odds ratio	CI	*p*
Introduction new crisis intervention track (after)	1.318	[0.016, 106.452]	.901
Time period	0.849	[0.778, 0.922]	<.001
Introduction new crisis intervention track × Time period	1.037	[0.776, 1.369]	.798
Percent of female patients	58.249	[0.122, 30538.547]	.198
Mean age at admission	0.937	[0.803, 1.090]	.400
First harmonic (sine)	1.098	[0.742, 1.627]	.639
First harmonic (cosine)	0.624	[0.451, 0.859]	.004

**Figure 1.**
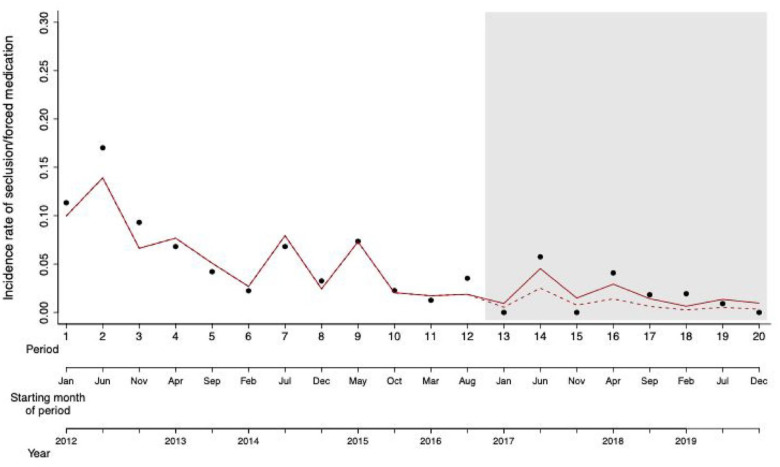
Scatter plot of the incidence rate of seclusion and/or forced medication over the 8 years (20 time periods) of the study. The shaded area represents the time periods when the new crisis intervention track was introduced. The solid red line represents the variation in the incidence rate as predicted by the model. The dashed red line represents the counter-factual scenario whereby incidence rate after intervention (*introduction new crisis intervention trac*k) is predicted based on data collected before the intervention using a model including only the time period and seasonality but not the intervention.

The data show that already before establishing the special crisis intervention track the incidence rate of seclusion and/or forced medication had reached a relatively low level. For a visual representation of the data see Figure 1.

Our sensitivity analyses yielded qualitatively similar results. For further information consult Supplemental Tables S2–S4.

Models exploring how different pairs of covariables explain the variation in the incidence rate of seclusion and/or forced medication were ranked according to their AIC and are presented in Supplemental Table S1. The best model included sex ratio and mean HoNOS item 2 and produced results qualitatively similar to the primary model.

**Figure 2.**
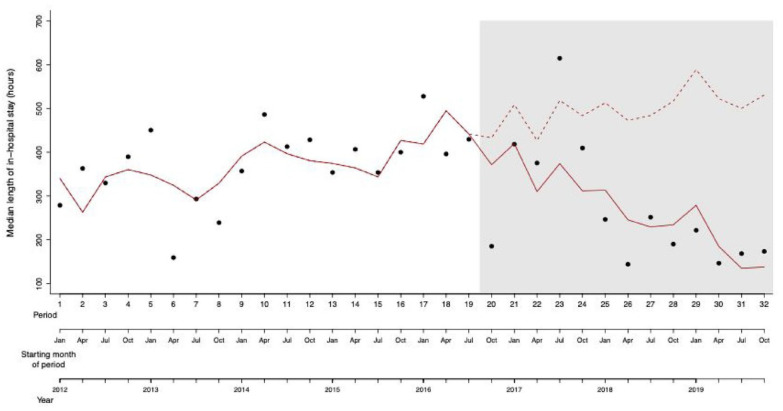
Scatter plot of the median length of in-hospital stay over the 32 time periods of the study. The grey area represents the time periods when the new crisis intervention track had been introduced. The solid red line represents the variation in the median length of in-hospital stay as predicted by the model. The dashed red line represents the counter-factual scenario whereby length of in-hospital stay after intervention is predicted based on data collected before the intervention using a model including only the time period, but not the intervention.

Table 3 shows the coefficients associating the median length of in-hospital stay (in hours) with the intervention (before/after introduction of the new crisis intervention track), the time period and their interaction, the mean patient age and the sex ratio. The best fitting model to explain the variation in the median length of in-hospital stay was a linear model assuming normal distribution of the dependent variable. The data show a significant interaction effect of the intervention and time (Introduction of the new crisis intervention track × Time; *p* = .002), which is indicative of a change in slope in the length of in-hospital stay in relation to time, that is, the median length of in-hospital stay initially increased, then decreased after the introduction of the new crisis intervention track (Figure 2).

**Table 3.** Linear model investigating the variation in the median length of in-hospital stay over the 32 time periods (trimesters) of the study from January 2012 to December 2019. Explanatory factors are time period, intervention (before/after introduction new crisis intervention track), and their interaction.

Variable	Estimate	CI	*p*
Introduction new crisis intervention track (after)	491.085	[124.631, 857.540]	.014
Time period	6.357	[−1.369, 14.082]	.119
Introduction new crisis intervention track × Time period	−27.630	[−43.176, −12.084]	.002
Percent of female patients	−506.809	[−1100.290, 86.673]	.106
Mean age at admission	−10.758	[−29.606, 8.090]	.273

Models exploring how different pairs of covariables explain the variation in the incidence rate of seclusion and/or forced medication were ranked according to their AIC and are presented in Supplemental Table S5. The best model included mean age and mean HoNOS item 2 and produced results qualitatively similar to the primary model (Supplemental Figure F2).

## Discussion

In this 8-year, hospital-wide, longitudinal, observational study, we investigated possible effects of the introduction of the new crisis intervention track for PD patients in October 2016, which we hypothesize played a key role in optimizing the crisis treatment concept for PD patients at the Department of Adult Psychiatry, UPK Basel. More specifically, we examined a possible association between the newly introduced crisis interventions track on the one hand and the frequency of seclusion and/or forced medication as well as the median length of in-hospital stay in PD patients on the other.

By means of an interrupted time series analysis, we predicted—based on the data on restriction and length of in-hospital stay in PD patients between January 2012 and October 2016—their further development in the hypothetical case that no new crisis intervention track had been introduced, and then compared this prediction with the actual development. To our knowledge, this is the first program evaluation to address the effectiveness of a specialized crisis intervention ward for PD crisis treatment over an 8-year observation period, which allows a reliable assessment of the time course before and after the “intervention,” that is, the introduction of the new crisis intervention track.

We examined the inpatient population of PD patients in a large psychiatric university hospital that has a mandate to admit all patients from the canton of Basel-Stadt. Therefore, it can be assumed that our analysis is an almost complete reflection of the general pool of PD patients, in particular of all patients requiring inpatient crisis intervention with coercion, and that the results of our study are generalizable. Further strengths of this study are the high quality of the data due to prospective electronic documentation and the long observation period over 8 years (2012–2019), which levels out seasonal effects and leads to a large number of patient cases examined. Moreover, the study reports findings using advanced statistical analyses that are supported by the results of the sensitivity analyses.

We found no evidence that the introduction of the new crisis intervention track for PD patients led to a reduction in the incidence rate of seclusion and/or forced medication in PD patients. However, the data show that the incidence of coercive measures in this population was already continuously decreasing between 2012 and 2016. This finding is consistent with other observational studies that showed a significant and continuous decline in coercion for all patients of the same psychiatric clinic in the years following the introduction of an open-door policy in 2010, the latter being hypothesized by the authors as the main reason for the declining incidence of restraint (Hochstrasser et al., 2018; Jungfer et al., 2014). The frequency of involuntary admission has decreased over the course of the observation period (8.1% of all PD patients in 2012 vs. 3.5.% in 2019). Again, this development can be well explained by the introduction of an open-door policy, as in this context also the indication for involuntary admissions has been examined more strictly. Our analysis shows that by the time the new crisis intervention track was introduced in October 2016, the frequency of seclusion and/or forced medication had already reached a very low level with little potential for further reduction. After the introduction of the new crisis intervention track for PD patients, coercive measures remained at this very low level. Therefore, we speculate that the lack of observable impact of the new crisis intervention track on coercion measures may be due to a floor effect rather than due to an actual absence of influence, especially since the effectiveness of such crisis interventions tracks—in particular the systematic admission of PD patients to a crisis intervention ward specially designed for this purpose—for the reduction of restraint in PD treatment has already been demonstrated in previous publications (Steinert et al., 2008; Steinert et al., 2009). Unlike in our setting, in these publications the introduction of the crisis intervention track was not preceded by the implementation of a hospital-wide open-door policy. This could explain the—compared to our patient population—high percentage of seclusions at the time of the intervention (15.0%), which dropped to 2.7% after the intervention. In contrast, in our hospital the percentage of restraints in the population we studied was 1.5% before the intervention and remained at a similarly low level after the intervention. This indicates that a sustainable reduction of coercive measures in the treatment of PD requires institutional adjustments on different levels that work synergistically: on hospital level, an overall conscious and selective use of coercive measures is of importance—which we have been practising in our hospital since 2010 in the context of the newly introduced open-door policy. At the same time, a diagnosis-specific range of therapies is required that enables therapeutic interventions to be fine-tuned to the patients’ needs—as in the case of PD through a crisis intervention track and a specialized crisis intervention ward.

Our second analysis shows that the median length of in-hospital stay of PD patients significantly decreased after the introduction of the new crisis intervention track for these patients. At this point, it is important to emphasize that all patients with PD main diagnosis were considered for this study—also those who were insufficiently stabilized on the crisis intervention ward and transferred to another ward so that treatment could be continued there. This is why the preferential admission of PD patients to the intervention ward (where therapy is time-limited to a max. of 7 days) alone does *not* explain the overall shortened length of stay. Our finding is in line with previous investigations on general mental health crises: A recent publication on psychiatric crisis management in adolescents (12–18 years) demonstrates a shorter length of in-hospital stay after the establishment of a crisis intervention ward, which, unlike in our case, was not specifically designed for particular diagnoses (Otterson et al., 2021). In a systematic review, data from 12 studies across six countries with a total of 67,505 participants were analyzed regarding the effectiveness of short-stay inpatient psychiatric crisis wards. In this review, meta-analyses indicated—among other findings—that both length of emergency department stays and number of subsequent inpatient admissions could be significantly reduced by the establishment of such wards (Anderson et al., 2022). One of the studies included in the review showed, similar to our study, a significant decrease of the length of in-hospital stay in patients presenting to an emergency department with behavioral health complaints which the authors put down to the introduction of a new crisis management concept offering patient-centred assessments and active crisis management for up to 48 hr (Lester et al., 2018).

In summary, this second result supports our hypothesis that a track concept for PD patients in crisis and in particular the selective use of short-term inpatient treatment on crisis intervention wards is of great relevance for the effectiveness of PD treatment. This becomes all the more important as we are looking at a patient clientele that is known not to benefit from long-term inpatient treatment in the vast majority of cases (Euler et al., 2018). Patients for whom the time-limited crisis intervention of a maximum of 7 days on the crisis ward doesn’t lead to a sufficient clinical response and to whom an outpatient setting is not yet appropriate, are transferred to other wards for a continuation of inpatient treatment. Hence, there is no risk that the limited treatment duration on the crisis ward might negatively affect the clinical treatment outcome of PD patients. This is supported by the observation that there was no noticeable increase in re-admissions and adverse events (e.g., suicide after discharge) in clinical routine reporting.

Interestingly, between January 2012 and September 2016, that is, before the introduction of the new crisis intervention track for PD patients, the median length of in-hospital stay for these patients continuously increased. This is likely a reflection of the fact that the mental health care system in Basel-City at that time did not have sufficient capacity to cope with the complex demands that PD presented. We hypothesize that the lack of an individualized treatment concept for PD at the time may have had a self-reinforcing effect, in such a way that unnecessarily long stays led to an exacerbation of the disease, which in turn resulted in further lengthy inpatient stays. We do not consider this circumstance to be specific to Basel or a particular institution, but assume that it is very widespread. It illustrates once again that the treatment of PD requires special clinical sensitivity—to which we hope to contribute with this paper.

### Limitations

The present study has a number of methodological limitations. Due to its observational design without comparison group, it is not possible to prove true causality between the decrease in median length of in-hospital stay and the introduction of the new crisis intervention track. Although from a clinical point of view the latter is likely the most reasonable and relevant explanation for the observed changes, further research with control groups, preferably in a randomized controlled trial design, is encouraged to verify our findings and to dismiss the possibility that they are mostly due to a secular and intervention-independent trend. Such a study, on the other hand, is difficult to conduct for ethical reasons and might have its own limitations.

Several features of the present study speak in principle for the generalizability of our results: the long observation period of 8 years, the large number of patient cases examined in total and the fact that the hospital where the study was performed has a treatment mandate for all psychiatric patients of the Canton of Basel-City, that is, no preselection of uncomplicated patient cases has to be assumed. On the other hand, the frequency and nature of coercive measures differ according to the local and national setting regarding healthcare systems and legal framework. Also, the feasibility of establishing a crisis intervention ward and its exact design depend strongly on the institutional possibilities of each hospital. In this way, the crisis intervention ward at the Department of Adult Psychiatry, UPK Basel, has characteristics that may not be easily generalizable, such as the introduction of further training on PD-specialized therapy for the staff of the new ward or its location on the campus of the University Hospital Basel (USB). This physical distance to the campus of the Department of Adult Psychiatry, UPK Basel, might increase the threshold for spontaneous transfers to wards with less specialized and longer treatment settings. Also, the somatic-medical environment is likely to contribute to destigmatizing psychiatric illnesses and crises (Huber et al., 2015; Sowislo et al., 2017; Steiger et al., 2022), which could have an additional deescalating effect. All these potential confounders should be specifically addressed in future research.

Furthermore, clinical routine data were used for the current analyses. Although they were prospectively entered in an electronic documentation system and it is known that data quality and completeness are sufficient for scientific use, only basic clinical data were available and some clinically desirable information, for example, psychopathology, adherence to treatment or history of coercion, was not available. However, this is a common problem and similar limitations exist for other research on comparable topics (Huber et al., 2016).

## Conclusion

In this 8-year, hospital-wide, longitudinal study, a crisis intervention track for PD patients was implemented. Since then, PD patients in crisis have been systematically admitted to a specialized short-term crisis ward. This is in line with the crisis management strategy recommended in the Swiss therapy guidelines. The intervention was accompanied by a stable low incidence of coercive measures and led to a clinically relevant and statistically significant reduction of the median length of in-hospital stay of these patients. In the authors’ view, this provides further evidence for the superiority of specialized short-term inpatient crisis management in PD with rapid reintegration into outpatient settings.

## Supplemental Material

sj-docx-1-isp-10.1177_00207640241277161 – Supplemental material for Specialized short term crisis intervention for patients with personality disorder: Effects on coercion and length of stay Supplemental material, sj-docx-1-isp-10.1177_00207640241277161 for Specialized short term crisis intervention for patients with personality disorder: Effects on coercion and length of stay by Lisa L. Dinsenbacher, Lukas Imfeld, Fabrice Helfenstein, Julian Moeller, Undine E. Lang and Christian G. Huber in International Journal of Social Psychiatry

## References

[bibr1-00207640241277161] Anderson K. Goldsmith L. P. Lomani J. Ali Z. Clarke G. Crowe C. Jarman H. Johnson S. McDaid D. Pariza P. (2022). Short-stay crisis units for mental health patients on crisis care pathways: Systematic review and meta-analysis. BJPsych Open, 8(4), 1–14. 35876075 10.1192/bjo.2022.534 PMC9344431

[bibr2-00207640241277161] Deuschle M. Scheydt S. Hirjak D. Borgwedel D. Erk K. Hennig O. Heser M. Pfister M. Leweke M. F. Meyer-Lindenberg A. (2020). Track-Behandlung in der psychiatrie: das ZI-track-modell zur Überwindung von Sektorengrenzen. Der Nervenarzt, 91(1), 50–56. 30941457 10.1007/s00115-019-0704-8

[bibr3-00207640241277161] Euler S. Dammann G. Endtner K. Leihener F. Perroud N. A. Reisch T. Schmeck K. Sollberger D. Walter M. Kramer U. (2018). Borderlinestörung: Behandlungsempfehlungen der SGPP. Swiss archives of neurology, psychiatry and psychotherapy, 5, 135–143.

[bibr4-00207640241277161] Gunderson J. G. (2009). Borderline personality disorder: A clinical guide. American Psychiatric Pub.

[bibr5-00207640241277161] Gunderson J. G. Weinberg I. Choi-Kain L. (2013). Borderline personality disorder. Focus, 11(2), 129–145.

[bibr6-00207640241277161] Hirjak D. Gass P. Deuschle M. Leweke F. M. Boehringer A. Schenkel N. Borgwedel D. Heser M. Breisacher A. Meyer-Lindenberg A. (2020). The CIMH track concept in the treatment of psychotic disorders. Der Nervenarzt, 91(3), 233–242. https://doi.org/https://doi.org/10.1007/s00115-019-0711-9 30976829 10.1007/s00115-019-0711-9

[bibr7-00207640241277161] Hochstrasser L. Fröhlich D. Schneeberger A. Borgwardt S. Lang U. Stieglitz R.-D. Huber C. (2018). Long-term reduction of seclusion and forced medication on a hospital-wide level: implementation of an open-door policy over 6 years. European Psychiatry, 48(1), 51–57. https://doi.org/https://doi.org/10.1016/j.eurpsy.2017.09.008 29331599 10.1016/j.eurpsy.2017.09.008

[bibr8-00207640241277161] Huber C. G. Schneeberger A. R. Kowalinski E. Fröhlich D. von Felten S. Walter M. Zinkler M. Beine K. Heinz A. Borgwardt S. Lang U. E. (2016). Suicide risk and absconding in psychiatric hospitals with and without open door policies: A 15 year, observational study. Lancet Psychiatry, 3(9), 842–849. 27477886 10.1016/S2215-0366(16)30168-7

[bibr9-00207640241277161] Huber C. G. Sowislo J. F. Schneeberger A. R. Bodenmann B. F. Lang U. E. (2015). Empowerment – ein Weg zur Entstigmatisierung. Swiss Archives of Neurology and Psychiatry 166, 224–231.

[bibr10-00207640241277161] Jungfer H. A. Schneeberger A. R. Borgwardt S. Walter M. Vogel M. Gairing S. K. Lang U. E. Huber C. G. (2014). Reduction of seclusion on a hospital-wide level: successful implementation of a less restrictive policy. Journal of Psychiatric Research, 54, 94–99. 24726637 10.1016/j.jpsychires.2014.03.020

[bibr11-00207640241277161] Lang U. E. Borgwardt S. Walter M. Huber C. G. (2017). Einführung einer »Offenen Tür Politik« - Was bedeutet diese konkret und wie wirkt sie sich auf Zwangsmaßnahmen aus? Recht & Psychiatrie, 35(2), 72–79.

[bibr12-00207640241277161] Lang U. E. Gaupp R. Huber C. (2022). Offene Psychiatrie dank Trackkonzept. Schweizerische Ärztezeitung, 103, 714–715.

[bibr13-00207640241277161] Lang U. E. Walter M. Borgwardt S. Heinz A. (2016). About the reduction of compulsory measures by an “Open Door Policy”. Psychiatrische Praxis, 43(6), 299–301. 27607432 10.1055/s-0042-111032

[bibr14-00207640241277161] Lester N. A. Thompson L. R. Herget K. Stephens J. A. Campo J. V. Adkins E. J. Terndrup T. E. Moffatt-Bruce S. (2018). CALM interventions: Behavioral health crisis assessment, linkage, and management improve patient care. American Journal of Medical Quality, 33(1), 65–71. 28693348 10.1177/1062860617696154

[bibr15-00207640241277161] McDowall D. McCleary R. Bartos B. J. (2019). Interrupted time series analysis. Oxford University Press.

[bibr16-00207640241277161] Organization W. H. (1992). The ICD-10 classification of mental and behavioural disorders: clinical descriptions and diagnostic guidelines. World Health Organization.

[bibr17-00207640241277161] Otterson S. E. Fristad M. A. McBee-Strayer S. Bruns E. Chen J. Schellhause Z. Bridge J. Murphy M. A. (2021). Length of stay and readmission data for adolescents psychiatrically treated on a youth crisis stabilization unit versus a traditional inpatient unit. Evidence-Based Practice in Child and Adolescent Mental Health, 6(4), 484–489.

[bibr18-00207640241277161] Paris J. (2004). Is hospitalization useful for suicidal patients with borderline personality disorder? Journal of Personality Disorders, 18(3: Special issue), 240–247. 15237044 10.1521/pedi.18.3.240.35443

[bibr19-00207640241277161] R Development Core Team. (2022). R: A language and environment for statistical computing. In R version 4.2.0 (2022-04-22) R Foundation for Statistical Computing. https://www.r-project.org

[bibr20-00207640241277161] Sollberger D. Lang U. (2013). Psychiatrie mit offenen Türen: Teil 1: Rational für Türöffnungen in der Akutpsychiatrie. Der Nervenarzt, 85, 1–7. 10.1007/s00115-013-3769-9 23538944

[bibr21-00207640241277161] Sollberger D. Lang U. (2014). Psychiatry with open doors. Part 2: Therapeutic challenges. Der Nervenarzt, 85(3), 319–325. 23579876 10.1007/s00115-013-3770-3

[bibr22-00207640241277161] Sowislo J. F. Lange C. Euler S. Hachtel H. Walter M. Borgwardt S. Lang U. E. Huber C. G. (2017). Stigmatization of psychiatric symptoms and psychiatric service use: a vignette-based representative population survey. European Archives of Psychiatry and Clinical Neuroscience, 267(4), 351–357. 27761652 10.1007/s00406-016-0729-y

[bibr23-00207640241277161] Steiger S. Moeller J. Sowislo J. F. Lieb R. Lang U. E. Huber C. G. (2022). Approval of coercion in psychiatry in public perception and the role of stigmatization [Original Research]. Frontiers in Psychiatry, 12, 819573. https://www.frontiersin.org/articles/10.3389/fpsyt.2021.819573 35069299 10.3389/fpsyt.2021.819573 PMC8777226

[bibr24-00207640241277161] Steinert T. Eisele F. Goeser U. Tschoeke S. Uhlmann C. Schmid P. (2008). Successful interventions on an organisational level to reduce violence and coercive interventions in in-patients with adjustment disorders and personality disorders. Clinical Practice and Epidemiology in Mental Health, 4(1), 1–6. 19014698 10.1186/1745-0179-4-27 PMC2596103

[bibr25-00207640241277161] Steinert T. Eisele F. Göser U. Tschöke S. Solmaz S. Falk S. (2009). Quality of processes and results in psychiatry: Decreasing coercive interventions and violence among patients with personality disorders by implementation of a crisis intervention ward. Gesundheitsökonomie & Qualitätsmanagement, 14(01), 44–48. https://doi.org/https://doi.org/10.1055/s-2008-1027448

[bibr26-00207640241277161] Steinert T. Martin V. Baur M. Bohnet U. Goebel R. Hermelink G. Kronstorfer R. Kuster W. Martinez-Funk B. Roser M. (2007). Diagnosis-related frequency of compulsory measures in 10 German psychiatric hospitals and correlates with hospital characteristics. Social Psychiatry and Psychiatric Epidemiology, 42(2), 140–145. 17180296 10.1007/s00127-006-0137-0

