# Supplementary material for: Specialized short term crisis intervention for patients with personality disorder: Effects on coercion and length of stay
Source: Int J Soc Psychiatry. 2024 Sep 4;70(8):1516–24. doi: 10.1177/00207640241277161 (PMC11528961; doi:10.1177/00207640241277161)
Supplement: sj-docx-1-isp-10.1177_00207640241277161 – Supplemental material for Specialized short term crisis intervention for patients with personality disorder: Effects on coercion and length of stay [file sj-docx-1-isp-10.1177_00207640241277161.docx]

**Table S1.** Performance statistics and comparisons of the null model (intercept only), the model including no covariates, and models including different pairs of covariates to explain variation in the occurrence rate of seclusion and/or forced medication. The model that performs best is the one with the smallest AIC, namely the model including sex ratio and mean HoNOS item 2 at admission.

|  | Model | Residual deviance | df | AIC | BIC | Delta AIC |
| --- | --- | --- | --- | --- | --- | --- |
|  | Sex-ratio & HoNOS item 2 | 12.49 | 11 | 77.54 | 85.10 | 0.00 |
|  | Sex-ratio & HoNOS item 1 | 12.71 | 11 | 77.76 | 85.31 | 0.21 |
|  | Mean age & HoNOS item 1 | 12.87 | 11 | 77.92 | 85.47 | 0.38 |
|  | Mean age & HoNOS item 2 | 13.85 | 11 | 78.90 | 86.46 | 1.36 |
|  | Sex-ratio & Percent law enforcement | 13.43 | 12 | 82.62 | 90.58 | 5.08 |
|  | Sex-ratio & Mean age at admission | 13.47 | 12 | 82.66 | 90.62 | 5.12 |
|  | Sex-ratio & Percent rooms occupied | 13.65 | 12 | 82.85 | 90.81 | 5.30 |
|  | Sex-ratio & Percent PD patients | 14.02 | 12 | 83.21 | 91.18 | 5.67 |
|  | Mean age & Percent law enforcement | 14.39 | 12 | 83.58 | 91.54 | 6.04 |
|  | Mean age & Percent rooms occupied | 14.75 | 12 | 83.94 | 91.91 | 6.40 |
|  | Mean age & Percent PD patients | 15.12 | 12 | 84.32 | 92.28 | 6.78 |
|  | Null model, intercept only | 67.83 | 19 | 123.02 | 124.02 | 45.48 |

AIC is a unitless measure describing how well a model describes the data, while accounting for the model’s complexity (i.e., the number of predictors). The smaller the AIC, the more parsimonious the model, i.e., the better the fit to the data and the smaller the number of predictors.

**Table S2.** Generalized linear model investigating the variation in the rate of occurrence of seclusion/forced medication from 2012 to 2019 assuming Poisson distribution of the error and including the logarithm of the population size per time-period as an offset. Effects of the explanatory variables are assessed as odds-ratios, i.e., coefficients applying to the probability of occurrence of seclusion/forced medication. The AIC of the full model was 83.9. The AIC of the null model (intercept only) was 125.86.

|  | Variable | Odds ratio | CI | p |
| --- | --- | --- | --- | --- |
|  | Ward opening (after) | 1.339 | [0.018, 101.033] | 0.893 |
|  | Time period | 0.848 | [0.779, 0.919] | <0.001 |
|  | Ward opening x Time period | 1.037 | [0.779, 1.363] | 0.797 |
|  | Percent of female patients | 51.403 | [0.134, 21818.185] | 0.196 |
|  | Mean age at admission | 0.936 | [0.808, 1.083] | 0.379 |
|  | First harmonic (sine) | 1.095 | [0.749, 1.606] | 0.639 |
|  | First harmonic (cosine) | 0.623 | [0.457, 0.847] | 0.003 |

**Table S3.** Linear model (normal distribution) investigating the variation in the probability of occurrence of seclusion/forced medication from 2012 to 2019 as estimated by the ratio of the number of cases of seclusion/forced medication over the population size per time-period. Effects of the explanatory variables are assessed as coefficients applying to the incidence rate of seclusion/forced medication. The AIC of the full model was -84.7. The AIC of the null model (intercept only) was -65.8.

|  | Variable | Estimate | CI | p |
| --- | --- | --- | --- | --- |
|  | Ward opening (after) | -0.065 | [-0.206, 0.076] | 0.337 |
|  | Time period | -0.010 | [-0.015, -0.005] | <0.001 |
|  | Ward opening x Time period | 0.008 | [-0.001, 0.017] | 0.092 |
|  | Percent of female patients | 0.048 | [-0.327, 0.423] | 0.786 |
|  | Mean age at admission | -0.003 | [-0.011, 0.005] | 0.420 |
|  | First harmonic (sine) | 0.002 | [-0.018, 0.021] | 0.855 |
|  | First harmonic (cosine) | -0.023 | [-0.043, -0.003] | 0.026 |

**Table S4.** Generalized linear model investigating the variation in the probability of occurrence of seclusion/forced medication over the 20 time periods of the study from January 2012 to December 2019 when using a different rule to assign seclusion/forced medication events to a given time-period. Effects of the explanatory variables are assessed as odds-ratios, i.e., coefficients applying to the probability of occurrence of seclusion/forced medication. The AIC of the full model was 80.7. The AIC of the null model (intercept only) was 123.3.

|  | Variable | Odds ratio | CI | p |
| --- | --- | --- | --- | --- |
|  | Ward opening (after) | 1.113 | [0.014, 89.930] | 0.962 |
|  | Time period | 0.854 | [0.784, 0.927] | <0.001 |
|  | Ward opening x Time period | 1.039 | [0.777, 1.373] | 0.791 |
|  | Percent of female patients | 23.984 | [0.050, 12251.683] | 0.314 |
|  | Mean age at admission | 0.945 | [0.810, 1.100] | 0.467 |
|  | First harmonic (sine) | 1.253 | [0.844, 1.872] | 0.265 |
|  | First harmonic (cosine) | 0.567 | [0.407, 0.784] | <0.001 |

**Table S5.** Performance statistics and comparisons of the null model (intercept only), the model including no covariates, and models including different pairs of covariates to explain variation in median length of in-hospital stay. The model that performs best is the one with the smallest AIC, namely the model including mean age and mean HoNOS item 2 at admission.

|  | Model | Residual deviance | df | AIC | BIC | Delta AIC |
| --- | --- | --- | --- | --- | --- | --- |
|  | Mean age & HoNOS item 2 | 1.67 | 24 | 356.47 | 366.28 | 0.00 |
|  | Sex-ratio & HoNOS item 2 | 1.93 | 24 | 360.75 | 370.56 | 4.28 |
|  | Sex-ratio & HoNOS item 1 | 2.01 | 24 | 362.06 | 371.87 | 5.59 |
|  | Mean age & HoNOS item 1 | 2.20 | 24 | 364.75 | 374.56 | 8.28 |
|  | Sex-ratio & Mean age at admission | 2.16 | 26 | 385.67 | 395.93 | 29.20 |
|  | No covariates | 2.48 | 28 | 386.16 | 393.49 | 29.69 |
|  | Sex-ratio & Percent rooms occupied | 2.24 | 26 | 386.75 | 397.01 | 30.28 |
|  | Sex-ratio & Percent PD patients | 2.25 | 26 | 386.97 | 397.23 | 30.50 |
|  | Sex-ratio & Percent law enforcement | 2.25 | 26 | 386.97 | 397.23 | 30.50 |
|  | Mean age & Percent law enforcement | 2.31 | 26 | 387.86 | 398.12 | 31.39 |
|  | Mean age & Percent rooms occupied | 2.35 | 26 | 388.39 | 398.64 | 31.92 |
|  | Mean age & Percent PD patients | 2.36 | 26 | 388.57 | 398.83 | 32.10 |
|  | Null model, intercept only | 4.66 | 31 | 400.65 | 403.58 | 44.18 |

AIC is a unitless measure describing how well a model describes the data, while accounting for the model’s complexity (i.e., the number of predictors). The smaller the AIC, the more parsimonious the model, i.e., the better the fit to the data and the smaller the number of predictors.

**S6: Documentation and management of clinical data**

Clinical and treatment data were continuously documented with the Medfolio software (current version: 2.2.0.2085; NEXUS AG, Villingen-Schwenningen, Germany) and extracted with HCe Analytics software (Business Intelligence Connector 3; BIC 3) for medical controlling (TIP Management AG, Dübendorf, Switzerland). Data on age, gender, diagnoses according to the International Classification of Diseases, 10th revision (ICD-10), type of admission and coercive measures were documented by the responsible psychiatrists. The same applies for the severity of aggression/overactivity (item 1) and of self-harm/suicidality (item 2) which was operationalized through the admission score on the Health of the Nations Outcome Scales (HoNOS) (21) as part of the standard admission assessments on all wards at the Department of Adult Psychiatry, UPK Basel.

With regard to coercive measures, both forced isolation and forced medication were considered: Forced isolation is referred to as "seclusion" in the present study and is defined as the involuntary confinement of a person locked alone in a room; forced medication is defined as oral ingestion or intramuscular administration of a drug against the patient's will, using restraint or strong psychological pressure, involving at least three staff members. Both the simultaneous use of seclusion and forced medication and the use of only one of the two measures at a time were considered equally as one incidence of coercion. Data on physical restraint using belts or straps were not available for the current analyses as this measure is not used at our hospital. Involuntary hospitalization is a further coercive measure, but since only public health officers and local authorities are allowed to initiate an involuntary hospitalisation in the canton of Basel-City, this coercive measure cannot be directly influenced by changes in hospital policies. Therefore, involuntary hospitalization was not chosen as an outcome parameter. It has however been included in the sample description and as a potential confounder in the multivariate analyses.

Concerning the secondary objective, the variable “in-hospital stay” describes the total number of hours spent at UPK, regardless of the ward or any transfers between wards.

**S7: Statistical analyses in detail**

To assess the effect of the intervention (*introduction of a new crisis intervention track for patients with PD*) on the incidence rate of seclusion and/or forced medication, we used a multivariable segmented generalized linear model with binomial distribution and logit link-function and with the number of cases of seclusion and/or forced medication per time period (number of events) over the number of inpatients with PD (number of trials) as the dependent variable. Intervention (factor: before/after introduction of the new crisis intervention track), time period and their interaction were included as explanatory variables. The time factor describes the temporal dynamics of the rate of coercive measures independently of the intervention. The intervention factor tests for an effect of the introduction of a new crisis intervention track on the overall rate of coercive measures independently of time (“level change” hypothesis). Their interaction describes a change in the temporal dynamics in the occurrence of coercive measures after the introduction of the new crisis intervention track (“impact” hypothesis). The model further included Fourier terms (one pair of sine/cosine over a 12-month period) to account for seasonality, as well as mean patient age at admission and sex ratio (% of females) as covariables. The demographic covariables were selected a priori to limit the number of factors in the model and avoid overparameterization and based on their suspected effect on the incidence of seclusion and/or forced medication.

To assess the effect of the intervention on the median length of in-hospital stay, we used a multivariable segmented generalized linear model with the median length of in-hospital stay of PD patients (in hours) as the dependent variable assuming gamma distribution with a log link-function. Intervention (factor: before/after introduction of the new crisis intervention track), time period and their interaction were included as explanatory variables. Here again, the time factor describes the temporal dynamics of median length of in-hospital stay independently of the intervention. The intervention factor tests for an effect of the introduction of a new crisis intervention track on the overall median duration of in-hospital stays independently of time (“level change” hypothesis). Their interaction describes a change in the temporal dynamics in the median length of in-hospital stay after the introduction of the new crisis intervention track (“impact” hypothesis). The model further included Fourier terms (one pair of sine/cosine over a 12-month period) to account for seasonality, as well as mean patient age at admission and sex ratio (% of females) as covariables. Again, the demographic covariables were selected a priori to limit the number of factors in the model and avoid overparameterization and based on their suspected effect on the median length of in-hospital stay.

We assumed that the primary endpoint followed a binomial distribution. However, a better fit to the data may be achieved using different distributions and link-functions. Consequently, as sensitivity analyses, we ran models using alternative distributions and/or link-functions. Models with similar distribution but different link functions were compared using Akaike Information Criterions (AIC). The model fit of all models was assessed using visual inspection of QQ-plots, leverage plots and plots of the residuals against the predicted values.

We further explored the sensitivity of the statistical models to the inclusion of other pairs of covariables. Besides age and sex ratio, our pre-selected covariables were aggression/overactivity at admission (mean HoNOS item 1), self-harm/suicidality at admission (mean HoNOS item 2), percentage of occupied rooms in the hospital as an indicator for staff capacity and percentage of patients presented to the hospital by law enforcement authorities (involuntary admission). We predefined age and sex ratio as covariables with highest clinical relevance. Nevertheless, to explore which of the covariables best explain variation in the incidence rate of seclusion and/or forced medication and because the number of covariables we could test without overparameterizing our model was limited by the number of patients/time periods included in our study, we ran all possible models including either mean age or sex ratio plus one of the other covariables. Models were compared using AIC.

We a priori assumed that the median length of in-hospital stay followed a gamma distribution, i.e., a right-skewed distribution. However, a better fit to the data may be achieved using different distributions and link-functions. Consequently, we also ran models using alternative distributions and/or link-functions. Models with similar distribution (but different link functions) were compared using AICs. The model fit of all models was assessed using visual inspection of QQ-plots, leverage plots and plots of the residuals against the predicted values. Here, we only present results for the best fitting model.


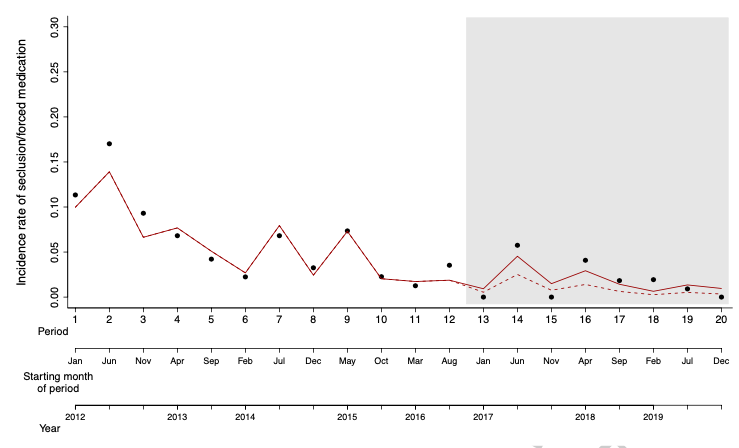
**Figure S1F.** Scatter plot of the incidence rate of seclusion and/or forced medication over the 8 years (20 time periods) of the study. The shaded area represents the time periods when the new crisis intervention track was introduced. The solid red line represents the variation in the incidence rate as predicted by the model. The dashed red line represents the counter-factual scenario whereby incidence rate after intervention (*introduction new crisis intervention trac*k) is predicted based on data collected before the intervention using a model including only the time period and seasonality but not the intervention.
